# The significance of lactate-to-serum albumin ratio in pediatric sepsis

**DOI:** 10.1097/MD.0000000000044773

**Published:** 2025-09-26

**Authors:** Lifen Yuan, Yingmei Li

**Affiliations:** a Department of Pediatrics, The Second Affiliated Hospital of Zhejiang University, Linping Campus, Hangzhou, China.

**Keywords:** children, lactate, lactate-to-serum albumin ratio, sepsis, serum albumin

## Abstract

This study aimed to evaluate the predictive value of the lactate-to-albumin ratio (L/A ratio) for sepsis in children at admission. This retrospective study involved all children with sepsis admitted to our pediatric intensive care unit from January 2021 to January 2024. Clinical data of included patients were collected through our hospital’s databases. The Pediatric Risk of Mortality and Pediatric Index of Mortality II scores were calculated, and lactate, albumin, and L/A ratio were measured within 24 hours of admission. Multivariate analysis was performed to evaluate the independent association of the L/A ratio and the risk of sepsis on the day of admission. This study included 100 children (50 with sepsis, 50 non-sepsis). The sepsis group had a higher level of lactate and L/A ratio.The serum albumin was considerably lower compared with the non-sepsis group. Logistic regression analysis suggested that the lactate, serum albumin, L/A ratio, Pediatric Risk of Mortality score, and Pediatric Index of Mortality II score are independent risk factors for sepsis. Moreover, the area under the curve of the lactate, serum albumin, and L/A ratio is 0.76, 0.69, and 0.85, respectively. Our findings highlight the importance of the L/A ratio as a robust marker for evaluating children with sepsis.

## 1. Introduction

Pediatric sepsis is a significant global health issue and a leading cause of death among children.^[1,2]^ Despite significant advancements in antibiotics and supportive care, pediatric sepsis remains difficult to treat, with high morbidity and mortality rates worldwide.^[3]^ As sepsis is still leading causes of morbidity and mortality, early diagnosis and timely antimicrobial therapy are critical for prompt clinical decision-making. Blood cultures are the gold standard for diagnosing sepsis; however, their clinical utility is compromised by lengthy processing times and the potential for false negatives, particularly in pediatric patients.^[4]^ Therefore, it is crucial to develop effective biomarkers for the early diagnosis and prognosis of sepsis.

The body produces lactate under hypoxic conditions or when tissue perfusion is inadequate. Several recent studies have shown that serum lactate concentrations were elevated during sepsis, and serum lactate is a gold-standard marker for sepsis whereby increased levels of lactate in sepsis patients are correlated with poor prognosis.^[5,6]^ In contrast, Serum albumin has also been reported to be a biomarker of prognosis in patients with sepsis.^[7]^ Currently, there is a lack of research support for the value of lactate-to-albumin ratio (L/A ratio) in predicting sepsis prognosis. This study focuses on pediatric patients with sepsis to investigate the predictive value of early L/A, serum lactate, and serum albumin levels in determining sepsis outcomes.

## 2. Methods

### 2.1. Study population

This retrospective study examined 50 children with sepsis admitted to the pediatric intensive care unit (PICU) of the first hospital in Linping district between January 2021 and January 2024. The inclusion criteria were: children aged over 1 month and under 14 years; diagnosed with sepsis requiring PICU admission; and obtaining parental consent. The exclusion criteria were: children with severe hepatic and renal dysfunction; children receiving long-term glucocorticosteroids; and incomplete clinical data. Sepsis patients were classified based on established criteria for sepsis.^[8,9]^ A cohort of matched 50 healthy individuals was also involved to serve as a control group. This study was approved by the medical ethics committee of the first hospital of Linping district (approval number: Linping2023044). Because of the retrospective nature of this study, the requirement for informed consent was waived.

Two routinely used illness severity indicators are the Pediatric Index of Mortality II (PIM II) and the Pediatric Risk of Mortality (PRISM).^[10,11]^ PRISM score and PIM II were automatically calculated within 24 hours of admission.

The primary outcome measure was the occurrence of death during hospital admission. The secondary outcome measures included the length of PICU stay and the need and duration of mechanical ventilation.

### 2.2. Data collection

Demographic data and clinical and laboratory parameters of enrolled patients were collected from the electronic medical record system in the hospital. Body temperature, systolic blood pressure, and heart rate are collected. Complete blood count results, C-reactive protein (CRP), and procalcitonin (PCT) levels were recorded. Whole blood was collected for testing within the first 3 hours after admission. Furthermore, 2 mortality predictive scores were calculated to determine the severity of disease including PRISM and PIM II.

### 2.3. Data analyses and statistics

Statistical analysis was performed using SPSS 26.0 software (IBM Corp., Armonk). Since the continuous variables were not normally distributed, they are expressed as median with interquartile ranges. Comparisons between groups were performed using the non rank sum test Mann–Whitney *U* test. Categorical variables are expressed as cases (%), and comparisons between groups were performed using the *χ*^2^ test. In the multifactor logistic regression model, the risk factors in the univariate analysis were selected as covariates. A receiver operating characteristic curve (ROC) was used to analyze the predictive value of L/A, serum lactate, and albumin on the first day of admission for sepsis. Differences were considered statistically significant at *P* < .05.

## 3. Results

### 3.1. Baseline comparisons

The study included 50 children diagnosed with sepsis and 50 healthy children as the control group. Their main demographic and clinical characteristics are shown in Table 1. With regard to the laboratory data, the levels of CRP, PCT, white blood cell, lactate, and L/A in the sepsis group were significantly higher (*P* < .05). Conversely, the albumin levels were significantly lower than those of the control group (*P* < .05). Otherwise, there was no significant difference in sex, age, body mass index, systolic blood pressure, neutrophil, and lymphocyte level between the sepsis and non-sepsis groups.

**Table 1:** Baseline characteristics of studied population.

	Sepsis (n = 50)	Non-sepsis (n = 50)	*P*
Sex (male, %)	27 (54)	22 (44)	.488
Age (yr)	9.2 (8.4–10.3)	8.8 (8.2–10.1)	.356
BMI (kg/m^2^)	24.24 ± 2.26	23.78 ± 2.75	.656
SBP (mm Hg)	99 (85–108)	94 (86–99)	.332
CRP (mg/L)	40.26 ± 18.37	5.34 ± 3.86	.001
PCT (ng/mL)	5.53 ± 2.24	0.23 ± 0.18	.001
Neutrophil (×10^9^/L)	8.46 ± 2.34	7.63 ± 1.28	.168
Lymphocyte (×10^9^/L)	3.65 ± 1.42	3.12 ± 1.46	.245
WBC (×10^9^/L)	15.42 ± 3.49	10.25 ± 2.32	.001
Lactate (mmol/L)	5.78 ± 2.21	1.5 ± 0.46	.001
Albumin (g/L)	23.52 ± 2.36	27.43 ± 1.56	.001
L/A (mmol/L)	0.24 ± 0.12	0.06 ± 0.03	.001
Mechanical ventilation, n (%)	18 (36%)	–	–
PICU stay (days)	10.5 (9–12)	–	–
PRISM	10 (8–12)	–	–
PIM II	8.5 (7.5–10.2)	–	–

Data are presented as mean ± standard deviation, n (%), or median (interquartile range).

BMI = body mass index, CRP = C-reactive protein, L/A = lactate-to-albumin, PCT = procalcitonin, PICU = pediatric intensive care unit, PIM II = Pediatric Index of Mortality II, PRISM = Pediatric Risk of Mortality, SBP = systolic blood pressure, WBC = white blood cell.

### 3.2. Survivor versus non-survivor analysis

The clinical and demographic parameters of the survivors and non-survivors were shown in Table 2. Non-survived group had significantly higher PRISM and PIM II score; longer PICU stay; and increased mechanical ventilation need compared with survived group. Besides, the levels of CRP, PCT, lactate, and L/A in the sepsis group were significantly higher. In contrast, the levels of albumin were markedly lower than those of the control group.

**Table 2:** Comparison between survivors and non-survivor groups regarding demographic data and clinical characteristics.

	Study groups (n = 50)		*P*-value
	Survivors (n = 39)	Non-survivors (n = 11)	
Sex (male, %)	17 (43.6)	4 (36.4)	.453
Age (yr)	9.2 (8.6–10.1)	7.8 (7.2–8.6)	.168
BMI (kg/m^2^)	23.1 (20.5–25.4)	22.1 (20.1–24.5)	.328
SBP (mm Hg)	99.2 (92.4105.8)	93.1 (86.8–99.5)	.248
CRP (mg/L)	41.26 ± 19.37	76.34 ± 18.86	.001
PCT (ng/mL)	5.53 ± 3.24	7.26 ± 1.28	.001
Neutrophil (×10^9^/L)	9.66 ± 2.14	10.63 ± 1.38	.168
Lymphocyte (×10^9^/L)	3.16 ± 1.28	3.32 ± 1.36	.468
WBC (×10^9^/L)	15.28 ± 3.42	15.78 ± 2.42	.827
Lactate (mmol/L)	3.56 ± 0.85	9.54 ± 2.28	.001
Albumin (mmol/L)	24.62 ± 1.25	21.18 ± 1.26	.001
L/A (mmol/L)	0.15 ± 0.06	0.38 ± 0.16	.001
Mechanical ventilation, n (%)	7 (18.0%)	11 (100%)	.001
PICU stay (days)	8.6 (7.2–9.4)	11.6 (10.5–13.8)	.001
PRISM score	6.8 (5.9–8.2)	14.3 (13.2–16.8)	.001
PIM II score	4.8 (4.2–5.9)	15.2 (13.8–16.2)	.001

Data are presented as mean ± standard deviation, n (%), or median (interquartile range).

BMI = body mass index, CRP = C-reactive protein, L/A = lactate-to-albumin, PCT = procalcitonin, PICU = pediatric intensive care unit, PIM II = Pediatric Index of Mortality II, PRISM = Pediatric Risk of Mortality, SBP = systolic blood pressure, WBC = white blood cell.

### 3.3. Logistic regression

A logistic regression analysis was conducted, which excluded white blood cell counts due to a *P*-value >.05. However, the logistic regression showed that the CRP, PCT, PRISM, PIMII, Lactate, Albumin, and L/A (*P* < .05) were independent risk factors for sepsis. Therefore, these factors were included as predictive indicators (Table 3).

**Table 3:** Multivariate regression analysis of risk factors for sepsis.

Factors	*β*	Wald	OR	95% CI	*P*
CRP	0.191	9.638	1.68	1.26–4.58	.001
PCT	0.005	7.253	2.16	1.46–4.63	.007
PRISM	0.220	5.345	2.65	1.45–3.63	.021
PIM II	0.027	7.502	3.64	1.64–5.45	.001
Lactate	0.056	4.579	1.35	1.13–2.08	.006
Albumin	1.492	28.994	1.46	1.20–1.85	.011
L/A	1.321	15.229	3.25	2.23–5.43	.001

CI = confidence interval, CRP = C-reactive protein, L/A = lactate-to-albumin, OR = odds ratio, PCT = procalcitonin, PIM II = Pediatric Index of Mortality II, PRISM = Pediatric Risk of Mortality.

### 3.4. ROC analysis

**Figure 1.**
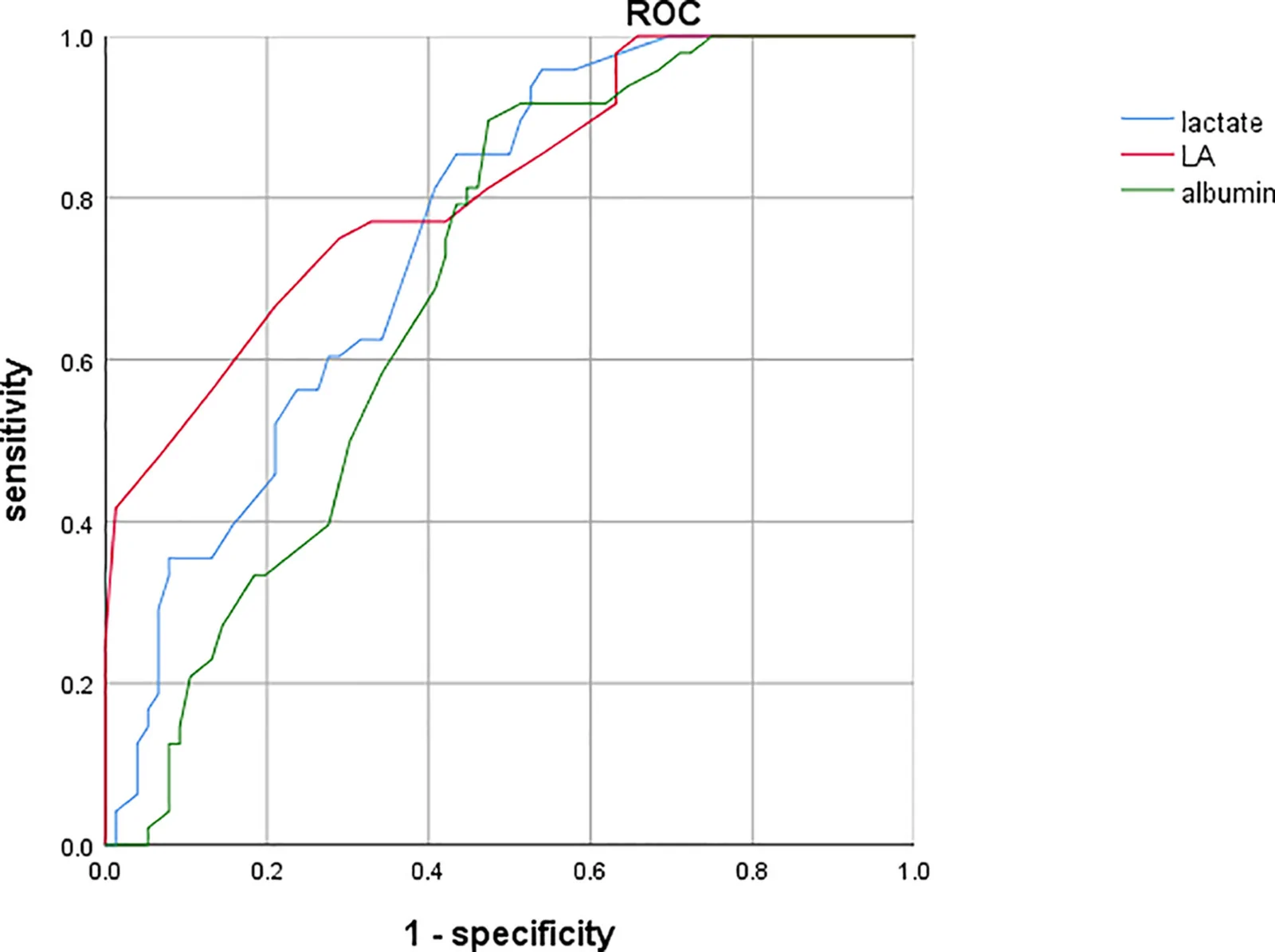
ROC curve to evaluate the diagnosis for sepsis. ROC = receiver operating characteristic.

We performed an ROC curve to evaluate the value of the lactate, albumin, and L/A index in the diagnosis of children with sepsis. As is shown in Figure 1, the area under the curves for the lactate, albumin, and L/A index were 0.760 (95% confidence interval [CI]: 0.664–0.880, and the specificity and sensitivity were 84.29% and 88.36%), 0.690 (95% CI: 0.615–0.853, the specificity and sensitivity were 78.65% and 84.16%), and 0.850 (95% CI: 0.774–0.892, the specificity and sensitivity were 88.25% and 86.16%), respectively.The final model demonstrated excellent fit (Hosmer-Lemeshow *χ*^2^ = 6.84, *P* = .55) with no evidence of multicollinearity (all variance inflation factor < 2.0).

## 4. Discussion

Sepsis is a syndrome marked by various physiological, pathological, and biochemical abnormalities. It triggers an uncontrolled inflammatory response in the host, which can lead to potentially fatal dysfunction of multiple organs. Inflammation and oxidative stress drive the pathophysiological process.^[7,8]^ The prevalence of sepsis is increasing, with sepsis emerging as a primary cause of mortality in PICU admissions.^[12]^ Hence, the detection of biomarkers to foresee sepsis risk can enhance early-stage clinical intervention, diagnosis, and treatment, ultimately enhancing children’s prognosis and quality of life. In our research, we analyzed the relationship between lactate-to-serum albumin ratio and sepsis on the first day of admission among children. Our findings indicate that lactate, albumin, and the L/A are independent risk factors for sepsis and are valuable for predicting its development.

Lactic acid is produced in response to hypoxia or inadequate tissue perfusion. Glucose undergoes anaerobic glycolysis. Pyruvate dehydrogenase cannot convert pyruvate into acetyl-CoA in time. Pyruvate begins to be produced in large quantities. At the beginning of the reaction, 2 molecules of adenosine triphosphate will be consumed. Eventually producing 4 molecules of adenosine triphosphate, which are catalyzed by lactate dehydrogenase and reduced to lactic acid, which begins to accumulate in the body.^[13]^ Studies have shown that elevated lactate levels are associated with mortality and are widely used in the early diagnosis, management, and risk stratification of patients with septic shock.^[14,15]^ However, elevated lactate is also affected by many factors, including drugs, liver and kidney dysfunction, diabetic ketoacidosis, cachexia, etc.^[16,17]^ The latest sepsis guidelines use lactate ≥ 4 mmol/L as a reference for poor prognosis in patients with septic shock.^[14]^ In our study, non-survived sepsis patients had a higher lactate; this is consistent with the conclusions of the guidelines. Therefore, lactate elevation can be used as an indicator to predict the prognosis of sepsis, and clinicians should pay close attention to it.

Human serum albumin as the most abundant protein in human blood plasma serves many physiological functions. The main functions include: regulating constant plasma osmotic pressure, transport function, oxidative decomposition to provide energy when the body is severely consumed, maintaining acid–base balance, functional barrier of vascular endothelial surface, antioxidant, and participation in coagulation function.^[18,19]^ The functions of albumin synthesis are also affected by various factors, including inflammation, malnutrition, and cirrhosis. Serum albumin, as an acute phase negative protein, can be used as a prognostic biomarker for sepsis patients.^[20,21]^ Our study found that serum albumin is a significant predictor of sepsis; non-survivors exhibited lower levels of serum albumin, reflecting its biological functions.

The L/A ratio is a better prognostic marker of multi-organ failure and mortality in septic patients. Additionally, a normal or low L/A ratio is linked to a more favorable prognosis.^[22,23]^ Some studies have shown the L/A ratio to be a better predictor of mortality in septic patients.^[24,25]^ Our findings were consistent with the mortality rates reported by other studies; moreover, L/A may be used as a prognostic indicators in sepsis patients.

This single-center retrospective study has several limitations. Large-sample studies are needed to increase the level of evidence for the relationship between sepsis and L/A. Beside, while our use of healthy controls provided a clear baseline for biomarker levels, this design choice may overestimate the discriminative power of the L/A ratio in clinical practice where differentiation between sepsis and other critical illnesses is often required. Future multicenter studies should include 3 comparator groups: septic patients, critically ill non-septic patients, and healthy controls to better evaluate the ratio’s true diagnostic specificity.

## 5. Conclusions

Our study indicates that lactate, albumin, and the L/A ratio may serve as early prognostic markers for sepsis. However, further studies are required to confirm this hypothesis.

## Author contributions

**Formal analysis:** Lifen Yuan.

**Investigation:** Lifen Yuan.

**Methodology:** Yingmei Li.

**Project administration:** Lifen Yuan, Yingmei Li.

**Resources:** Lifen Yuan.

**Supervision:** Yingmei Li.

**Writing – original draft:** Lifen Yuan.

**Writing – review & editing:** Yingmei Li.
